# Non-communicable diseases in disasters: a protocol for a systematic review

**DOI:** 10.5249/jivr.v13i1.1512

**Published:** 2021-01

**Authors:** Elham Ghazanchaei, Iraj Mohebbi, Fatemeh Nouri, Javad Aghazadeh-Attari, Davoud Khorasani-Zavareh

**Affiliations:** ^*a*^ Social Determinants of Health Research Center, Urmia University of Medical Sciences, Urmia, Iran.; ^*b*^ Department of Health in Emergencies and Disasters, School of Public Health and Safety, Shahid Beheshti University of Medical Sciences, Tehran, Iran.; ^*c*^ Workplace Health Promotion Research Center, Department of Health in Emergencies and Disasters, School of Public Health and Safety, Shahid Beheshti University of Medical Sciences, Tehran, Iran.

**Keywords:** Crisis, Natural Disaster, Management, Non Communicable Diseases, Chronic illness

## Abstract

**Background:**

NCDs require an ongoing management for optimal outcomes, which is challenging in emergency settings, because natural disasters increase the risk of acute NCD exacerbations and lead to health systems’ inability to respond. This study aims to develop a protocol for a systematic review on non-communicable diseases in natural disaster settings.

**Methods:**

This systematic review protocol is submitted to the International Prospective Register of Systematic Reviews (Registration No. CRD42020164032). The electronic databases to be used in this study include: Medline, Scopus, Web of Science, Clinical Key, CINAHL, EBSCO, Ovid, EMBASE, ProQuest, Google Scholar, Cochrane Library (Cochrane database of systematic reviews; Cochrane central Register of controlled Trials). Records from 1997 to 2019 are subject to this investigation. Three independent researchers will review the titles, abstracts, and full texts of articles eligible for inclusion, and if not matched, they will be reviewed by a final fourth reviewer. The proposed systematic review will be reported in accordance with the reporting guideline provided in the Preferred Reporting Items for Systematic review and Meta-Analysis (PRISMA) statement. We select studies based on: PICOs (Participants, Interventions, Comparators, and Outcomes).

**Results:**

This systematic review identifies any impacts of natural disasters on patients with NCDs in three stages i.e. before, during and in the aftermath of natural disasters.

**Conclusions:**

A comprehensive response to NCD management in natural disasters is an important but neglected aspect of non-communicable disease control and humanitarian response, which can significantly reduce the potential risk of morbidity and mortality associated with natural disasters.

## Introduction

Disasters ar e serious events disturbing communities.^1^ In terms of medical aspects, these events cause numerous casualties and the high demand of medical care may require the enhancement of responders’ capacity for delivering timely and eﬀective services.^2,3^ Individuals with chronic conditions require special attention in planning, response, and recovery phases after natural disasters, given their unique needs for medication, medical equipment and continuing healthcare, and potential exacerbation of their condition that require resource-intensive management.^4^ Natural disasters can impact the public health infrastructure and the social protection systems essential for vulnerable populations. Patients with non-communicable diseases e.g. respiratory and cardiovascular diseases, cancer and diabetes, are among vulnerable groups in critical conditions, who are highly affected by natural disasters.^5^

Non-Communicable diseases (NCDs) require an ongoing management for optimal outcomes, which is challenging in emergency settings, since natural disasters increase the risk of acute exacerbation in the health of people with NCDs and decrease the health systems responsiveness.^6^ NCD Management in emergencies requires the inclusion of non-communicable disease care into standard operating procedures, which would facilitate horizontal and vertical integration to other aspects of relief efforts.^7,8^

Patients with chronic illnesses including those with cardiovascular diseases, diabetes, cancers, and respiratory conditions are of the most vulnerable populations in disaster settings, who face various problems after natural disasters.^9,10^ By the collapse in some medical care systems and overloaded operating hospitals and other medical centers, provision of services to chronic patients seems to be a critical concern.^11^

Inadequately managed chronic illnesses can present a threat to life and well-being of the community in the immediate wake of these disasters, but their treatment traditionally has not been recognized as a public health or medical priority.^12^ Many patients did not have their medications or medical supplies, and too many did not know the names of their illnesses or medications or how to access the information.^13^

A critical problem in the resulting health crisis is the inability of the displaced population to manage their chronic diseases.^14^ The Center for Disease Control and Prevention (CDC) reported that NCDs accounted for five of the six most commonly reported conditions after Hurricane Katrina.^15^ This leads to indirect causes of mortality and more complications up to 70%-90%, primarily due to the deterioration of life-threatening conditions and exacerbation of chronic diseases.^16^ After natural disasters, inadequate care and resources, and lack of continuity of care for chronic diseases such as cardiovascular diseases, asthma, diabetes, renal diseases have led to exacerbation of symptoms associated with increased morbidity and mortality among this population.^17^ However, non-communicable diseases have received little attention from human-rights organizations during the acute phase of crises and emergencies, and there is a need to refocus on emergency disaster systems in the 21st Century.^18^ More than 45% of evacuees did not carry their daily medicines with them, meaning that over two third of total medicines provided during the disaster response were used to treat chronic diseases.^19^ Patients with chronic diseases face many challenges and have different needs during and after natural disasters and medical care must be continued during and after natural disasters. Statistics on different diseases reveal that at the time of natural disasters, there are an increased number of hospital admissions of patients with at least one chronic disease. As an example, in Sichuan earthquake, patients with hypertension and those with diabetes, constituted 47% and 24%, respectively, of city hospital admissions.^20^ Despite the significance and the critical impact of natural disasters on patients with non-communicable diseases and the exacerbation of their symptoms, there are not enough studies on the issue.^21^ Disaster and crisis manuals and guidelines mainly focus on communicable diseases like Aleppo boil, measles, cholera and diarrhea; and among available research literature, there is a limited number of studies on the management of non-communicable diseases in emergencies.^22^ Several studies have been conducted on the effects of natural disasters on non-communicable diseases reporting the exacerbation of clinical effects and insufficient medical facilities and equipment to care for patients. Therefore, this study aims to obtain a systematic review protocol for non-communicable diseases in the natural disasters.

## Methods and Methods

This systematic review protocol is submitted to the International Prospective Register of Systematic Reviews. (http://www.crd.york.ac.uk/PROSPERO) (The registration number was: CRD42020164032). Preferred Reporting Items for Systematic review and Meta-Analysis Protocols (PRISMAP) will be applied to develop this review protocol.^23^

**Eligibility Criteria**

Applying a systematic review method, authors will investigate studies focused on non-communicable diseases in disasters from different aspects including epidemiological factors, risks, effects, interventions, patient needs, preparedness, as well as academic literatures from around the world. This study and its findings are intended to serve as a roadmap for future research in this area, by giving information on intervention development and policy change.

The target population is the group of patients with chronic diseases. The top four leading causes of death in patients with NCDs, which constitute the tenets of the WHO 2013-2020 NCD action plan, are: cardiovascular diseases, cancer, chronic respiratory disease and diabetes. The formal search strategy will be applied for relevant controlled vocabularies and free text synonymous words and phrases in concept mapping for health conditions including heart attack, myocardia, ischemia, acute coronary syndrome, stroke, hypertension, diabetes, cancer, Chronic Obstructive Pulmonary Disease(COPD) and asthma through advanced search syntax. Authors will search for possible relevant titles in the reference list of eligible studies. There will be no natural disaster location or natural disaster type limitation in our search. Also there is no restriction on the research study design. Research papers in English will be qualified. PICO (Patient/Population/Participant, Intervention, Comparison, Outcome) framework will be applied in formulating questions and facilitate the search strategy articulation.

**Participants**

The subject patients in the study are those with chronic diseases including cardiovascular and chronic respiratory diseases, diabetes and cancer who are affected by natural disasters. Subject to our unlimited survey in terms of the natural disaster location, the population (participants) may belong to developing or developed countries.

**Interventions**

The study will investigate the management of health service delivery to people with non-communicable diseases who are affected by natural disasters. There is no restriction on the type of natural disaster.

**Comparison**

The only comparison to be made in this study will be that of the impact of natural disasters on the provision of medical care services to patients with non-communicable diseases before, during and after natural disasters.

**Outcomes**

The study will classify findings based on the type of non-communicable diseases, the type of natural disasters, and the natural disaster occurrence-NCD condition relationship (NCDs before, during and after natural disasters). Finally, results concerning the clinical impacts and symptom exacerbations in patients with non-communicable diseases and models of service delivery to these patients during natural disasters as well as challenges and deficiencies in the study will be discussed.

**Information Sources and Search Strategy**

The electronic database search strategy will be adopted to gather relevant information from 1997 thru 2019 (reviews published before this period are likely to be out of date) using the following databases: Medline, Scopus, Web of Science, Clinical Key, CINAHL, EBSCO, Ovid, EMBASE, ProQuest, Google Scholar, Cochrane Library (Cochrane database of systematic reviews; Cochrane central Register of controlled Trials. Three independent reviewers will evaluate titles, abstracts and full texts of eligible articles for inclusion, and the final vetting is to be by the fourth reviewer, in case of discrepancies. The search process may be re-run and more studies may be retrieved for inclusion before the final analysis. The search strategy will be developed based on the MeSH terms and Key words related to natural disasters and non-communicable disease. The focus will be on the top four leading causes of NCD-related death, constituting the tenets of the WHO 2013-2020 Global NCD Action Plan i.e. cardiovascular and chronic respiratory diseases, cancer, and diabetes. The formal search strategy will be applied for relevant controlled vocabularies and free text synonymous words and phrases in concept mapping for health conditions including heart attack, myocardia, ischemia, acute coronary syndrome, stroke, hypertension, diabetes, cancer, COPD, asthma. The search strategy applied in electronic databases like the PubMed database is provided in Appendix 1.

**Data Collection and Extraction**

Reference lists of searched out articles will be examined to identify further studies. Also, bibliographies in systematic and non-systematic review articles will be investigated to identify relevant studies.

For any query pertaining to methodology, study outcomes and data collection, authors will contact reviewers. Abstracts and full texts of searched out manuscripts will be reviewed. Reference lists of systematic reviews and included studies will be screened and citation tracking will be conducted, wherever feasible. Multiple publications and overlaps will be identified, grouped and represented as a single reference. Database search results will be imported into the citation management program to aggregate relevant review articles and exclude duplicates. 

**Figure 1:**
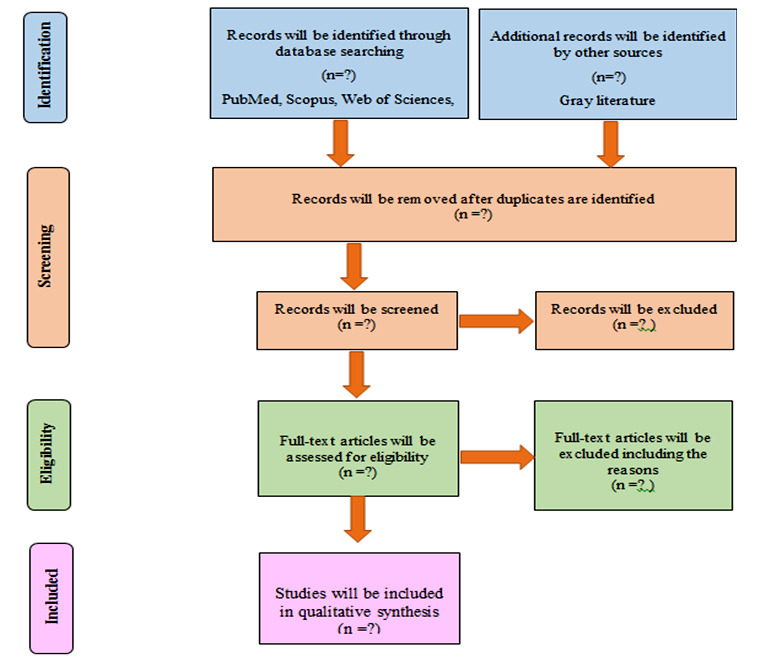
PRISMA diagram for study selection non-communicable diseases in natural disasters: A protocol for a systematic review.

Titles and abstracts of all reviews searched out from electronic databases will be imported into EndNote (EndNote X6) and duplicates will be excluded. Authors will search and review titles, abstracts and keywords. Duplicate references will be omitted, and afterwards two of the reviewers (EGh, FN) will screen the titles. Three independent researchers will evaluate titles, abstracts and full texts of eligible articles for inclusion (DKZ, EGH, FN), and there will be a final vetting by the fourth reviewer (IM), in case of discrepancies, who will check the results. The search process may be re-run and more studies may be retrieved for inclusion just before the final analysis. A predefined inclusion and exclusion criterion is to be used for assessment of the full texts of the remaining titles. The excluded studies will be listed in a table associated with the reason for exclusion. Data extraction will be processed electronically using a developed data abstraction form adapted from the Cochrane Public Health Group. By data extraction and assessment form, any information on aspects deemed necessary as per Methodological Expectations of Cochrane Intervention Reviews (MECIR) standards will be collected.^24^ The data abstraction form will be piloted on a random sample of five included articles, and modifications will be made as required, based on the team’s feedback. Full data abstraction will be started only when there is a sufficient agreement (i.e. The percentage of agreement >90 %). Data extraction for the literature review will be based on study goals, characteristics (e.g. the first author, the publication date), search strategy and terminology, to describe the review and its settings and timeframe policy. The process of selecting studies will be documented in a PRISMA flow chart. (Figure 1)

**Quality Assessment**

The researchers will evaluate the quality of selected articles based on valid checklists for study types. The quality assessment of observational studies such as cohort and cross-sectional articles will be carried out by strengthening the reporting of observational studies in epidemiology check list (STROBE).^25^ Based on this checklist, ranking scores are from 0 to 34 and studies will be classified into 3 groups based on their ranking score as follows: weak quality ranking score from 0 to 11; moderate quality ranking score from 12 to 22; and high quality ranking score from 23 to 34. The quality assessment of experimental studies will be carried out by transparent reporting of evaluations with nonrandomized designs (TREND).^26^ Based on this checklist, ranking scores are from 0 to 59 and studies will be classified into 3 groups based on their ranking score as follows: weak quality ranking score from 0 to 21; moderate quality ranking score from 22 to 41; and high quality ranking score from 42 to 59. The quality assessment of qualitative studies will be carried out using the Critical Appraisal Skills Programme (CASP). ^27^ Based on this checklist, ranking scores are from 0 to 10 and studies will be classified into 2 groups based on their ranking score as follows: weak quality ranking score from 0 to 5; and high quality ranking score from 6 to 10. The quality assessment of the systematic reviews and meta-analyses will be carried out by the checklist for preferred reporting items for systematic reviews and meta-analyses (PRISMA).^23^ The checklist consists of 27 items and papers are reviewed for each item and marked either as implemented or not-implemented. In case of the absence of an item in a paper, it will be rated ZERO, and if the subject item exists in the paper, it will be rated ONE. When items are not as distinct, the unclear sections will be assessed several times until a precise interpretation is reached and a valid evaluation of the study is made. The risk of bias will be assessed using ROBIS Risk of Bias assessment tool.^23,28^

**Appendix 1:** Example of search strategy.

PubMed	(noncommunicable[Title/Abstract]) AND (disasters[Title/Abstract]) (disaster*[Title/Abstract] OR emergency*[Title/Abstract]) AND (noncommunicable[Title/Abstract]) (natural[Title] AND disaster*[Title]) AND (("noncommunicable"*)[Title] OR (chronic)[Title] OR ("illness"*)[Title] OR ("cancer")[Title] OR ("chronic")[Title] OR (disease*)[Title] OR ("lung"*)[Title] OR ("cardiovascular"*)[Title] OR ("pulmonary")[Title] OR (diabet*)[Title] OR (malignancy*)[Title]) (((((((("noncommunicable"[Title] AND disaster)[Title]) OR (("noncommunicable disaster"*)[Title])) OR ((chronic*)[Title])) AND ((disaster)[Title])) OR (("natural disasters")[Title])) OR ((disaster[Title] AND natural)[Title])) OR ((disasters[Title] AND natural)[Title])) OR (("natural disaster")[Title]) (((((((("noncommunicable"[Title] AND disaster)[Title]) OR (("noncommunicable disaster"*)[Title])) OR ((chronic*)[Title])) AND ((disaster)[Title])) OR (("natural disasters")[Title])) OR ((disaster[Title] AND natural)[Title])) OR ((disasters[Title] AND natural)[Title])) OR (("natural disaster")[Title]) ((non communicable*)[Title/Abstract]) AND ((emergence*)[Title/Abstract] OR (haz-ard)[Title/Abstract] OR (disaster*)[Title/Abstract] OR (natural disasters)[Title/Abstract] OR ("natural disaster")[Title/Abstract] OR (disaster[Title/Abstract] AND natural)[Title/Abstract] OR (disasters[Title/Abstract] AND natural)[Title/Abstract]) (("natural disasters")[Title/Abstract]) AND ((COPD*)[Title/Abstract] OR ("lung disease*")[Title/Abstract] OR (cancer*)[Title/Abstract] OR ("heart disease"*)[Title/Abstract] OR ("diabete"*)[Title/Abstract] OR ("chronic"*)[Title/Abstract]) ((natural disaster) OR (natural disasters) OR (disaster) OR ("natural disaster") OR (natural AND disaster) OR (natural AND disasters) OR ("emergence"*) OR ("hazard"*)) AND ((noncommunicable disease) OR (noncommunicable diseases) OR ("noncommunicable disease") OR (noncommunicable AND disease) OR (noncommunicable AND diseases) OR (chronic illness) OR (chronic AND illness) OR ("chronic illness") OR ("chronic"*))

## Results

The primary outcome of this study will be the identified needs of patients with chronic diseases such as diabetes, cardiovascular, hypertension, respiratory disease, and cancers that are critical before, during, and after natural disasters. Secondary outcome of the study will be the reported challenges in the process of health service provision to these patients. The following data will be extracted from the selected articles: general information (title, name of authors, year, research type, subject group (by disease type), classification (before, during and after natural disaster), and factors essential to follow a process of health service provision to patients with chronic illnesses in natural disasters.

**Acknowledgments**

This article is extracted from a PhD thesis on Health in Emergency and Disaster, with COI: IR.UMSU.REC.1398.228, the Research Center for Social Factors Effective on Health, Urmia University of Medical Sciences. We extend our special thanks to supervisors and advisors, who collaborated in this research.

**Abbreviations**

NCDs: Non-Communicable diseases

CDC: Center for Disease Control 

PICO: Patient/ Population/ Participants/ Problem, Intervention, Comparison, Outcome

COPD: Chronic Obstructive Pulmonary Disease

PROSPERO: Prospective Register of Systematic Reviews
